# Physical therapy students' perceptions of embedded medical librarians within evidence-based practice courses: a mixed-methods pilot study

**DOI:** 10.5195/jmla.2025.1977

**Published:** 2025-04-18

**Authors:** Lori Bolgla, Malorie Novak, Lachelle Smith

**Affiliations:** 1 lbolgla@augusta.edu, Professor in the Department of Physical Therapy; Kellett Chair in Allied Health Sciences, Augusta University, Augusta, GA; 2 malorie.novak@outlook.com, Associate Professor in the Department of Physical Therapy, Augusta University, Augusta, GA; 3 lsmith411@gatech.edu, Digital Learning Specialist Senior, Georgia Institute of Technology, Atlanta, GA

**Keywords:** Health Informatics, evidence based medicine, Physical Therapy, Academic Health Sciences Libraries

## Abstract

**Objective:**

Previous work within academic medical centers has indicated the potential value of embedded medical librarian programs within health sciences professional degree programs. This study sought to determine the perceived benefit that an embedded medical librarian (EML) provided to an evidence-based practice (EBP) course within an entry-level physical therapy degree program.

**Methods:**

Learners completed an anonymous survey at the end of an EBP course about the impact of the EML on the course and their own EML utilization. Frequency and percentages were calculated for quantitative data; qualitative data were analyzed using an iterative process for code development.

**Results:**

Forty (98%) learners completed the survey. Seventy-five point six percent of learners utilized the EML 1–2 times per class session and 31.7% outside of class sessions. Learners overwhelmingly “agreed” (53.7%) or “strongly agreed” (39.0%) that they would consult the EML for literature searches required in future courses. Seventy point seven percent “strongly agreed” that the EML improved their ability to conduct a literature search. All learners either “agreed” (43.9%) or “strongly agreed” (56.1%) that the EML added value to the course. Ninety point two percent considered the EML as an integral part of the course. Themes from the qualitative analysis agreed that the EML added value to the course and facilitated skills that would be useful throughout the curriculum.

**Conclusion:**

Learners believe that having an EML improves their ability to conduct a literature search. Providing learners with EML access during their education experience facilitates development of this skill. Early and continued instruction throughout the entry-level DPT curriculum in informatics ensures program compliance with accreditation standards.

## INTRODUCTION

The Commission on Accreditation in Physical Therapy Education (CAPTE) is the only body recognized by the US Department of Education and the Council for Higher Education Accreditation that can accredit entry-level Doctor of Physical Therapy (DPT) education programs. Accreditation attests that graduates of a PT program receive the quality of education required for entry-level PT practice. Accredited programs must meet all of the *Standards and Required Elements for Accreditation of Physical Therapist Education Programs* [1]. Required Element 7D40 states that entry-level DPT education programs instruct learners in the “use of health informatics in the health care environment” [1]. CAPTE defines health informatics as “the interdisciplinary study of the design, development, adoption, and application of IT-based innovations in healthcare services delivery, management, and planning” [2]. Failure to demonstrate evidence of fulfilling Element 7D40 has been identified as among the top ten areas of programmatic citation during accreditation review processes, which highlights the importance of creating and documenting initiatives related to Element 7D40.

In addition to expertise utilizing electronic health records, health informatics encompasses the ability to develop the skills necessary to locate quality information efficiently. A popular method for introducing information-seeking and evidence-based practice skills to health sciences students is the embedded medical librarian (EML) model. The EML model positions medical librarians into a proactive role by placing them virtually or physically in settings where learners are [3, 4]. This model promotes a proactive and participatory interaction, enabling an embedded librarian to work with faculty and learners across teaching, research, and clinical settings [4, 5].

Embedded medical librarian models have been implemented in academic medical centers in programs such as medicine, nursing, dentistry, pharmacy, public health, and music therapy [3, 6, 7, 8, 9]. Advantages of using the EML include ongoing collaboration to improve learners' information literacy and research skills [9]. By comparison, the depth of prior work examining the EML model within rehabilitation professions like PT and occupational therapy (OT) is limited. DaLomba et al. provided preliminary evidence to highlight the importance of using an EML to help learners in the rehabilitation sciences develop the skills necessary to locate the best evidence for enhancing clinical decision-making and improving patient outcomes [10]. Such investigations have not been conducted in DPT programs.

Since 2015, the College of Allied Health Sciences (CAHS) at August University has had an “embedded” librarian who conducted regular office hours and taught in a limited number of class sessions for all programs. However, the DPT program recognized the benefits of incorporating the EML model to meet accreditation standards and provide learners the best resource to seek evidence-based materials. A major change to the EBP course was having the EML play an active role in the delivery of the course content throughout the entire semester. Specifically, she worked with the course director to develop the course syllabus and all in-class literature search activities. The course syllabus included the location of the EML's designated office in the Department of Physical Therapy and specific office hours. The EML also was available by email and appointment.

The purpose of this study is to evaluate the perceived value of having an EML within this entry-level DPT program's EBP course. We hypothesize that learners would utilize the EML during and outside class sessions, consider the EML as an important part of the course, and report improved confidence in conducting literature searches.

## METHODS

### Class Instruction

The first lecture session focused on developing a searchable clinical question using the patient/problem, intervention, comparison, outcome (PICO) strategy, various databases to locate literature, and the use of Boolean operators. Instruction also included ways to maximize search success through the use and manipulation of medical subject headings (MeSH terms) and key words. All class sessions included an activity for learners to practice using the PICO strategy to locate evidence to answer a clinical question. The EML attended every class session and facilitated this activity. The activity began with a clinical scenario pertinent to PT practice. Learners analyzed the clinical scenario and placed key words in the PICO format. Next, they developed a searchable clinical question and used the PubMed database to identify an article to best answer the question. Afterward, the EML conducted a debriefing session that focused on the following: 1) important information included in the clinical scenario; 2) terms used in the PICO strategy; 3) format of the searchable clinical question; 4) use of MeSH terms and Boolean operators; and 5) locating the intended article.

Learners also completed a pass/fail activity outside of the regular class session that accounted for 10% of the overall course grade. They received 3 scenarios that they might encounter in clinical practice. One scenario required learners to locate an article that compared outcomes for individuals with patellofemoral pain who completed an intervention comprised of hip-based exercise to those who performed knee-based exercises. The next scenario was to determine the diagnostic accuracy of the Thessaly test for identifying a knee meniscal tear. The final scenario examined the ability of the Morse Fall Risk Scale to predict fall risks for patients in an acute care setting. Learners were graded on their ability to use the PICO strategy to develop a searchable clinical question. They also were graded on their search string (use of MeSH headings, key words, and Boolean operators) to locate an article to answer the question. All learners successfully completed the activity on their initial attempt.

### Course Evaluation Surveys

Forty-one first-year DPT learners (2020 summer semester) enrolled in their first EBP course (PTHP 7101, Research 1) were invited to participate. Participants were not required to sign an Augusta University IRB-approved informed consent document because this study was granted exempt status. Participants completed two anonymous surveys at the end of the 11-week course via QualtricsXM (Qualtrics, Provo, UT). Survey questions were adapted from Blake et al [11], who examined patron perception and utilization of an EML program. For the quantitative arm of this study, learners used a 4-point Likert scale (strongly disagree, disagree, agree, strongly agree) to answer four statements about the impact of the EML on the course (Appendix). They also answered two questions about the number of times they utilized the EML during a class session and outside a class session. For the qualitative arm of this study, learners were asked four open-ended questions about their beliefs regarding the following: 1) role of the EML; 2) value added by the EML; 3) skills gained from the EML; and 4) use of the EML for EBP (Appendix). The participants were informed prior to completing the surveys that investigators would not access survey results until all learners completed the course and received their final course grade.

### Data Analysis

For the quantitative data, frequency and percentages were calculated for each question. For the qualitative data, we used a qualitative thematic analysis with an iterative process for code development [12]. Three investigators (LB, MN, LS) independently reviewed all comments from the survey. They met and developed preliminary codes for the data as well as code definitions for a finalized codebook. The codebook was used to independently code each comment for a second time. The investigators met and finalized coding by consensus. Themes were verified by reviewing comments and assessing for disconfirming evidence.

## RESULTS

Ninety-eight percent (40/41) of the learners agreed to allow use of their responses for this study.

### Quantitative Data

Seventy-five point six percent of learners reported utilizing the EML 1–2 times per class session and 31.7% outside of class sessions (Figure). Most learners “agreed” (53.7%) or “strongly agreed” (39.0%) that they would consult the EML for literature searches required in future courses. Seventy point seven percent “strongly agreed” that the EML improved their ability to conduct a literature search. All learners either “agreed” (43.9%) or “strongly agreed” (56.1%) that the EML added value to the course. Ninety point two percent considered the EML as an integral part of the course.

**Figure 1:**
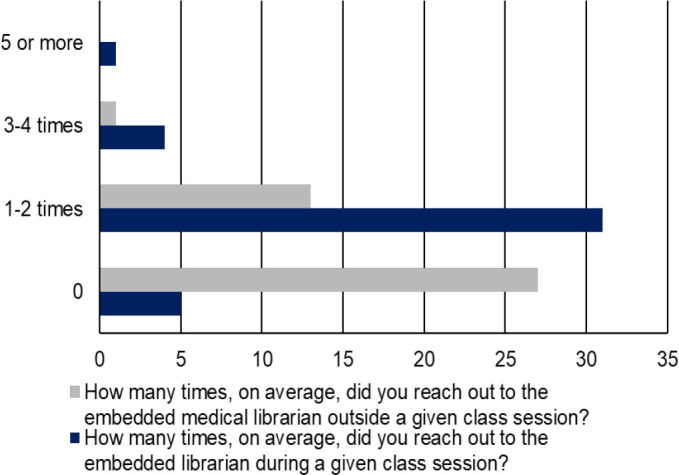
Frequency of the number of times learners contacted the embedded medical librarian over the course semester

### Qualitative Data

Within learners' responses to open response questions on the value of an embedded librarian within the course, three themes emerged: search process instruction, search process facilitation, and search process efficiency. The definition of each theme, along with exemplar quotes for each, are included in Table 1.

**Table 1:** Learners' perceived values of interacting with an embedded librarian within their physical therapy evidence-based practice course

Theme	Exemplar Quotes
Search Process Instruction: EML provided didactic knowledge to complete a literature review by teaching learners how to use PubMed, providing added technical expertise on the importance of EBP, guiding learners in formative activities, and providing repetition in search strategies and question formation.	*“She provided useful guidance and pertinent research search strategy information.”* *“…she effectively taught us the material and reviewed it many times so that we would understand it. She also provided us with a lot of helpful examples.”*
Search Process Facilitation: EML facilitated the actual search process by demonstrating how to use the PICO format, use MeSH terms, and construct search strategies.	*“…helped me to better understand how to narrow down my searches and formulate good questions for research.”* *“…provided guidance and pertinent research search strategy information.”*
Search Process Efficiency: EML improved the effectiveness by which the learners used tools to begin to incorporate relevant evidence into clinical decision-making.	*“She helped teach a more efficient way to search different sites to find research that will be the most beneficial for us.”* *“I learned how to access articles with a higher efficiency while the article were also at a much higher quality.”*

## DISCUSSION

The purpose of this study was to determine the value of having an EML in an EBP course for entry-level DPT learners. We hypothesized that learners would utilize the EML during and outside class sessions, consider the EML as a valuable part of the course, and report improved confidence with conducting literature searches. Findings from this study confirmed our hypotheses. The data gathered from this study's qualitative arm provided insight into learners' beliefs about the role of the EML, value of the EML, skills gained from the EML, and use of the EML in EBP.

The findings of this study align with prior evaluations of embedded librarian programs within health sciences degree programs. Blake et al [11] examined patron perception of EML across colleges (i.e., they did not examine by specific program) within a health sciences university. Ninety-four percent of their respondents either agreed or strongly agreed that they were satisfied with the EML service. Eighty-four percent of their respondents reported that they would seek EML help in the future, which is lower than the current study's 92.7% response rate. The ongoing interaction (i.e., development of a working relationship with the EML) between the EML and our learners most likely explained our higher response rate.

To date, we are not aware of any other entry-level DPT programs' use of the embedded librarian model and can attribute success in our program to various factors. Most importantly, the EML was actively engaged with the learners during most class sessions and played an integral role in content delivery. Anecdotally, as the course progressed, learners reached out to the EML more and more during each session. We believe that these in-class interactions fostered communication with the EML outside of class. Prior works have shown that EML visibility increases the likelihood of learners consulting the EML [13, 14]. Learners also reported more confidence in their search skills over the course of the semester. While many were familiar with databases like PubMed and Google Scholar, they were introduced to other databases more specific to PT practice like CINAHL Plus, SPORTDiscus, and ProQuest. We believe that the use of these databases contributed to learners developing more robust literature searchers [14]. Another important consideration was the administrative support provided by the Dean of the CAHS. The Dean ensured that the EML had dedicated office hours and encouraged all academic programs to integrate the EML into EBP activities. Although not a study focus, the EML was a member of the college-level faculty governance council. Her participation on this committee provided an additional avenue to foster collaboration with other faculty members. Programs that want to implement the embedded librarian model should consider the importance of communication to promote collaboration between the EML and instructors [3, 5]. They also should ensure adequate administrative support (e.g., designated office hours and physical space) and opportunities for engagement between faculty and learners [3, 5, 11, 13].

Our findings suggest that entry-level DPT programs looking to document their compliance with CAPTE's Element 7D40 (health informatics) competency could consider utilizing an embedded librarian program. Incorporating the EML model, especially within an EBP course, may provide learners with a means for developing the skills needed to be an efficient evidence-based practice clinician. Learners who develop fundamental information-seeking skills within early course sequences could then build on these skills by applying them within clinical decision-making settings later in their course sequence.

## LIMITATIONS

The study has limitations to address. First, our study lacked a pre-test/post-test design, thus prohibiting the ability to determine changes in the learners' attitude from interactions with the EML. Having data from this design could provide insight on the degree to which the embedded librarian model shaped learners' perception of the EML. Second, this study was designed to assess learners' perceptions, not actual attainment, of improved literature search skills. Although all learners successfully passed a single graded activity (e.g., using the PICO strategy to locate an article to answer a clinical question), it was not sufficient to comprehensively assess actual skill gains. Finally, this study only included a single cohort of DPT learners. The EML worked exclusively in the CAHS, which houses the DPT program and allowed our learners to have significant access to the EML throughout the course. We cannot determine the success of this model when using an EML that supports other disciplines like medicine, dentistry, and nursing. Together, these limitations impact the overall generalizability of study findings to other programs.

## FUTURE DIRECTION

The purpose of this pilot study was to determine the benefits of an EML having a significant role in delivering content in an entry-level DPT EBP course. Our findings suggest that having an EML integrated early into a DPT curriculum is valuable to the learners and that learners appreciate learning skills early in their course of study that they can utilize throughout the curriculum. Future studies should incorporate a pre-test/post-test design to examine changes in perceptions of the value of the EML and competency in conducting literature searches. Researchers also should examine the amount of EML interaction required to develop proficient search skills. Another need is to determine the extent that a cohort of entry-level DPT learners consult the EML in future evidence-based clinical courses. Generalizability would be improved by having other entry-level DPT programs replicate our survey. Finally, additional studies should determine the impact that the EML has on faculty productivity with respect to instruction and scholarship as well as contributions on college-level committees.

## Data Availability

Data associated with this article cannot be made publicly available because they contain personally identifiable information. Access to the data can be requested from the corresponding author and may be subject to IRB restrictions.

## Supplemental Files

**Appendix A:** Questions for the Study Quantitative Arm
