# Supplementary material for: Physical therapy students' perceptions of embedded medical librarians within evidence-based practice courses: a mixed-methods pilot study
Source: J Med Libr Assoc. 2025 Apr 18;113(2):143–7. doi: 10.5195/jmla.2025.1977 (PMC12058335; doi:10.5195/jmla.2025.1977)
Supplement: Supplementary file 1 — Appendix A: Questions for the Study Quantitative Arm [file jmla-113-2-143-s01.docx]

**QUESTIONS FOR THE STUDY QUANTITATIVE ARM**

1. How many times, on average, did you reach out to the embedded librarian during a given class session?

- 0
- 1-2 times
- 2-3 times
- 4 or more times

1. How many times, on average, did you reach out to the embedded medical librarian outside a given class session?

- 0
- 1-2 times
- 2-3 times
- 4 or more times

1. The embedded medical librarian added value to this course.

- Strongly disagree
- Disagree
- Agree
- Strongly agree

1. I will consult the embedded medical librarian in the future to assist with literature searches.

- Strongly disagree
- Disagree
- Agree
- Strongly agree

1. The embedded medical librarian was an integral part to this course.

- Strongly disagree
- Disagree
- Agree
- Strongly agree

1. The embedded medical librarian improved my ability to search evidence-based literature.

- Strongly disagree
- Disagree
- Agree
- Strongly agree

**QUESTIONS FOR THE STUDY QUALITATIVE ARM**

1. What was the role of embedded medical librarian?
2. Do you believe the embedded medical librarian added value to the course? If so, how?
3. What skills did you gain from this course through the embedded medical librarian?
4. How did you utilize the embedded medical librarian for evidence-based practice?
